# Lunatic Asylums in Ireland

**Published:** 1856-07-01

**Authors:** 


### LUNATIC ASYLUMS IN IRELAND.

Owing to the judgment pronounced by the Court of Queen's Bench in Dublin in January last, ignoring the validity of the appointment of chaplains by the Executive Government of Ireland in the Belfast District Asylum, and, as a consequence, in all the other public asylums in that country, it has become necessary for the Government to apply to the Legislature for power to make legal what has been solemnly declared to be illegal, as is palpable from the third clause of the Bill—"to Explain and Amend the Acts relating to Lunatic Asylums in Ireland" (No. 2)—now before us, which is as follows, viz.:—

"All appointments of officers for such Asylums, and all salaries of such officers fixed or granted, and all matters and things heretofore done by the Lord Lieutenant, or by the Lord Lieutenant and Council, with respect to such appointments, shall be and remain good, valid, and effectual, but subject to the powers and provisions hereinafter contained."

It remains to be seen if Parliament, before which the Bill is at present, will give its sanction to this *ex post facto* species of legislation, which of all others is to be looked upon with a very jealous eye, perverted as it might be to the most unconstitutional of purposes. The first clause of the Bill states that, "in citing this Act, it shall be sufficient to use the expression, 'the Lunatic Asylums (Ireland) Act, 1856.'" The second clause has reference to the interpretation of terms, viz:—"Asylums" shall mean "Asylums for the Lunatic poor." "Officer" shall mean and include "Managers, Chaplains, Physicians, Surgeons, Apothecaries, Matrons, Attendants, and Servants." This, we think, is a pretty enlarged, if not an offensive and levelling interpretation of the term "Officer." In the army, what would be thought of classing officers and privates in one category? In civil life and civil employment a little less insulting interpretation of officer and servant might have been adopted; and in no public institutions is it of more consequence to be rather on the particular than the loose side of using fit and proper terms, and observing due punctilio, than in establishments set apart for the treatment of the insane, where so much depends upon and is to be accomplished by the prestige of position, and the moral weight and influence such gives to those in their chief charge. And here we may state, that we think this is the fitting place again to express our decided opinion, that the time has now fully arrived when an effort should be made to give up the barbarous and unscientific nomenclature in common use as regards Institutions for the Insane and their inmates, and in their stead to adopt one more in harmony with the advancing intelligence of the age, and of a less offensive nature. The opportunity now presented by this Bill should not be lost by the Inspectors in Ireland, and others in authority, of disusing such terms as Lunatic, Manager, Matron, &c., all of which are highly objectionable, the first being absolutely wrong as applied to insanity, being under and affected by lunar influence and changes; the second leading to the notion that the medical man in charge—as, we presume, manager has reference to—has to break-in and restrain a number of animals instead of treating human beings under disease; and the third savouring strongly of the prison and the workhouse. Insane person, Hospital or Asylum for the Insane, Resident Physician or Medical Superintendent, and Superintendent of Females or Housekeeper, respectively, should be substituted for the above most objectionable terms. We therefore call the particular attention of the Inspectors, Drs. White and Nugent, to these points, in the full expectation that they will use their official influence in a matter of some importance, as we conceive this to be; for after all, there is something in a name, Shakspeare's dictum notwithstanding to the contrary that, "a rose by any other name would smell as sweet." Besides, we do not know if it have struck others as it has ourselves, that, if the term "manager" remain, any individual, any layman—educated or uneducated—a half-pay officer, or other totally unsuited person, might be appointed to an office no doubt intended, by a mental reservation, to be filled by a member of the medical profession, as it should, and as is and must be the case in this country, as well as on the continent and in America. Why, then, should so all-important a post of duty be left thus open to the caprice of any public functionary, no matter how high his office—even that of the Lord Lieutenant of Ireland, to be professionally filled up, who, by clause eight in the Bill, is to have the patronage of appointing "the Manager, Matron, and Visiting Physician?" The fourth clause stands thus—viz.,

"The Lord Lieutenant and Council shall, from time to time, in the case of every such Asylum established, or to be hereafter established, fix and determine the number and description of officers for every such Asylum."

According to the above clause, the Lord Lieutenant and Council might, if they pleased, appoint a posture master, a professor of gymnastics, a dancing master—and far more objectionable officials might be attached to asylums, if the course we have named could be considered exceptionable—in fact, any number of persons they pleased—so unlimited is the scope of this curiously framed clause; but the eighth one is, we presume, a set-off to it, being quite antagonistic in its powers—viz.,

"All officers of every such Asylum, other than the Manager, Matron, and Visiting Physician, shall be, from time to time, appointed by the Governors, and, whether now holding office or hereafter to be appointed, shall be removable at the pleasure of the Governors."

So that here we have, in one plan, the executive empowered to make appointments to an unlimited extent, and, in another, the power vested in the local boards of governors, of "removing all officers at their pleasure other than the Manager, Matron, and Visiting Physician"! The farce of blundering legislation could no farther go than this. For any public functionary or functionaries to be given so arbitrary a power as to remove an officer "at pleasure" is seriously to be deprecated, as it might be brought to bear in a manner nothing short of oppression, and yet without any remedy to the aggrieved party. No one could be considered in the light of a public servant who might at any time get his dismiss at the *pleasure* merely of a superior authority. Under such circumstances he is the servant of an irresponsible party *pro tanto*, and will be tempted accordingly to become a tool or worse in the hands of those who can place the screw upon him at any moment. This ought not so to be.

The remaining clauses of this Bill are as annexed—viz.,

The Governors of such Asylum shall, from time to time, subject to the approbation of the Lord Lieutenant in Council, fix the salaries to be paid to such officers respectively; but, if the Governors shall neglect so to do, or if the Lord Lieutenant in Council shall disapprove of the salary proposed for any officer, it shall be lawful for the Lord Lieutenant in Council to fix and determine the same.

It shall be lawful for the Governors, with the approval of the Lord Lieutenant in Council, from time to time, to alter the salaries to be paid to such officers respectively.

The Manager, Matron, and Visiting Physician of every such Asylum shall be, from time to time, appointed by the Lord Lieutenant, and, whether now holding office or hereafter to be appointed, shall be removable at the pleasure of the Lord Lieutenant.

It shall be lawful for the Governors, on the recommendation of the Inspectors of Lunatics, or of one of them, to direct that any officer who is incapable, from age, infirmity of mind or body, or otherwise, to discharge the duties of his office, shall be superannuated, and shall receive such yearly superannuation pension as, upon consideration of all the circumstances of each case, shall appear to be just, not exceeding such proportion of his salary and allowances as hereinafter mentioned (that is to say, for above fifteen and less than twenty years' service, a pension not exceeding two-thirds of his salary and allowances, and, for above twenty years' service, a pension not exceeding his salary and allowances).

The several salaries and superannuation pensions, now or hereafter to become payable, shall respectively be advanced, paid, presented for, and raised in like manner as any other moneys advanced or raised for supporting and maintaining such Asylums respectively, under the said recited Acts, or any of them.

Whereas pauper lunatics only can now, by law, be admitted into any such District Lunatic Asylums, and there are others of the industrious classes suffering from insanity who may be benefited by treatment in Lunatic Asylums, but whose relatives are unable to meet the expense of Private Asylums, and are not willing to accept gratuitous relief, and it is expedient that some provision should be made for such classes: Be it, therefore, enacted, that it shall be lawful for the Governors of any district Lunatic Asylum (subject to any orders to be made by the Lord Lieutenant in Council) to receive as inmates any persons not coming under the description of pauper lunatics (but to be treated in all respects as if pauper lunatics, clothing only excepted), on such terms as to payment or otherwise as the Governors shall deem proper, and the moneys so received as payment for such persons shall be applied to the support and maintenance of such Asylums.

It shall be lawful for the Lord Lieutenant in Council, from time to time, to make any general or special orders regulating the admission of such lunatics not coming under the description of paupers, or prohibiting the admission of such persons.

This Act and the said recited Acts shall be construed together as if one Act.

The superannuation clause is a redeeming one in this Bill, as the absence to the present time of any such provision for so meritorious a class of officers, has been long and justly considered a great grievance. The only suggestion we would venture to make on it, is, that those officers who had served "above twenty years" in the unceasingly onerous and responsible duties of an Asylum, should certainly be given the option of retiring on their well-earned pension, if they pleased, without requiring the preliminary "recommendation of the Inspectors to the Board of Governors" for such, on the plea of "incapability from age, infirmity of mind or body, or otherwise, to discharge the duties of his office." A little more liberality in this respect, would not have deteriorated from the purposes of the measure now under consideration.

The clause making provision for the reception of a class of patients not hitherto contemplated in the District Asylums of Ireland, appears open to many grave objections. Far be it from us to say a word against the relief of those, the subjects of mental derangement, who are neither paupers nor sufficiently in the enjoyment of the means of being placed in a private Asylum. We consider that the case of such unfortunately circumstanced persons is deserving of the utmost amount of sympathy. But to place this class, as is intended, in common with the ordinary inmates of a pauper Asylum, would, in our judgment, be nothing short of a "cruel kindness." If no other plan can be devised, an entirely separate and distinct portion of the District Asylums should be set apart for their care and treatment. Besides, it would lead to endless disturbance and jealousies, to mix up the pay and pauper inmates together. The former, and their friends, would still be soliciting and expecting additional indulgences and attendance—the latter, seeing, or suspecting even, anything of this kind, would not be slow in resenting it, and thus an under-current of dissatisfaction and envious feeling would be constantly at work, which would soon destroy that harmony of action so essential in any public establishment, especially those for the insane. The step, no doubt, is a benevolent one; but we are quite confident a wrong method has been taken to carry it into effect. No element of discord, however remote, should be introduced into the details of our public Asylums. The rights of the pauper insane should not, then, be trifled with, or experimented upon by the Legislature. At present, however, we can say nothing more on this important subject.

